# Hyperammonemic Encephalopathy: valproic acid-induced adverse reaction

**DOI:** 10.1192/j.eurpsy.2023.2235

**Published:** 2023-07-19

**Authors:** E. Gómez, L. Gallardo, R. Fernández, E. Talaya, L. Al Chaal, E. Rybak

**Affiliations:** 1 Psychiatry, General Hospital of Segovia (Spain), Segovia, Spain

## Abstract

**Introduction:**

Hyperammonemic encephalopathy is an unusual but fatal consequence of patients being treated with valproic acid (VPA). The most relevant clinical features in cases of valproic acid-induced hyperammonemia include confusion, lethargy, vomiting, and increased seizure frequency and may progress to stupor, coma, and lead to death in isolated cases. The causes are not fully elucidated, but studies suggest alterations in liver and kidney function with abnormalities in the urea cycle causing increased ammonium levels.

**Objectives:**

Clinical review and treatment approach for VPA-induced hyperammonemia encephalopathy.

**Methods:**

Clinical case and literature review.

**Results:**

A 23 - years - old male, admitted to the psychiatric unit for a psychotic episode in the context of drug use and associated affective symptoms. Treatment with antipsychotic (Risperidone 6mg per day) and mood stabilizer (valproic acid up to 1000/mg per day) was prescribed. After ten days of treatment, the patient started with low level of awareness and abnormal behaviour. Neurological examination showed marked somnolence, dysarthric language, unstable gait and behavioral alterations. In the physical examination the constants are stable with discrete tachycardia. Laboratory tests revealed hyperammonemia (609μg/dL), with normal liver function and serum concentration of total valproic acid was therapeutic (69mg/L). Brain computed tomography (CT) revealed no significant anomalies. Doctors initiated treatment with daily cleansing enema and VPA was suspended immediatly. After forty-eight hours the patient’s mental status gradually improved back to baseline and the ammonium levels were normalized in medical tests.

**Conclusions:**

Valproate-induced hyperammonemic encephalopathy is an unusual but serious complication. It is often underdiagnosed, with an unclearly incidence. The consequences of undertreatment can be potentially deadly. Clinical suspicion should be established in all patients with decreased level of consciousness in patients receiving VPA. Hyperammonemia can be asymptomatic in half of the cases and can occur in people with normal therapeutic doses and normal serum valproate levels. The mechanism of VPA-induced hyperammonemic encephalopathy is unclear. At present, it is thought to be primarily due to propionic acid, a metabolite of VPA, which inhibits an enzyme necessary for the elimination of ammonia in the urea cycle. In addition, VPA can raise plasma ammonia levels through interaction with carnitine, leading to increased renal excretion of carnitine. In terms of treatment, the main recommendations agree that discontinuation of valproate is the most effective therapy, followed by administration of lactulose to reduce ammonium levels. Carnitine supplementation may be useful in the following cases: for seizure disorders in children at risk of developing carnitine deficiency, in VPA poisoning and in VPA-induced hepatotoxicity.

**Disclosure of Interest:**

None Declared

